# Teleneonatal or routine resuscitation in extremely preterm infants: a randomized simulation trial

**DOI:** 10.1038/s41390-024-03545-1

**Published:** 2024-09-28

**Authors:** Samuel J. Gentle, Sarah G. Trulove, Nicholas Rockwell, Chrystal Rutledge, Stacy Gaither, Carrie Norwood, Eric Wallace, Waldemar A. Carlo, Nancy M. Tofil

**Affiliations:** 1 Department of Pediatrics, The University of Alabama at Birmingham, Birmingham, AL USA; 2 Department of Pediatrics, Yale School of Medicine, New Haven, Connecticut USA; 3 Department of Medicine, The University of Alabama at Birmingham, Birmingham, AL USA

## Abstract

**Objective:**

Teleneonatology, the use of telemedicine for newborn resuscitation and care, can connect experienced care providers with high-risk deliveries. In a simulated resuscitation, we hypothesized that teleneonatal resuscitation, compared to usual resuscitation, would reduce the no-flow fraction.

**Study design:**

This was a single-center, randomized simulation trial in which pediatric residents were randomized to teleneonatal or routine resuscitation. The primary outcome was no-flow fraction defined as time without chest compressions divided by the time during which the heart rate was <60. Secondary outcomes included corrective modifications of bag-mask ventilation and times to intubation and epinephrine administration.

**Results:**

Fifty-one residents completed the scenario. The no-flow fraction (median [IQR]) was significantly better in the teleneonatal group (0.06[0.05]) compared to the routine resuscitation group (0.07[0.82]); effect (95% CI): −16 (−43 to 0). Participants in the teleneonatal resuscitation group more frequently performed corrective modifications to bag-mask ventilation (60% vs 15%; *p* < 0.001). Time to intubation (214 s vs 230 s; *p* = 0.58) and epinephrine (395 s vs 444 s; *p* = 0.21) were comparable between groups.

**Conclusions:**

In this randomized simulation trial of neonatal resuscitation, teleneonatal resuscitation reduced adverse delivery outcomes compared to routine care. Further in hospital evaluation of teleneonatology may substantiate this technology’s impact on delivery outcomes.

**ClinicalTrials.gov ID:**

NCT04258722

**Impact:**

- Whereas telemedicine-supported neonatal resuscitation may improve the quality of resuscitation within hospital settings, unique challenges include the need for real-time, high-fidelity audio-video communication with a low failure rate.
- The no-flow fraction, which evaluates the quality of chest compressions when indicated, has been associated with survival in other clinical contexts. We report a reduction in no-flow fraction in neonatal resuscitations supported with telemedicine, in addition to improvements in the quality of neonatal resuscitation.
- Telemedicine-supported neonatal resuscitation may improve the quality of resuscitation within hospital settings without direct access to neonatologists.

## Introduction

Effective neonatal resuscitation requires the timely implementation of evidence-based guidelines from the American Academy of Pediatrics.^1^ Access to highly trained neonatologists, more experienced in neonatal resuscitation, improves outcomes in preterm infants as supported by prior analyses of neonatal outcomes by hospital level.^2,3^ Moreover, with the continued decline in access to level III delivery hospitals,^4^ the concurrent increase in risk for the delivery of preterm infants at lower-level facilities has been associated with increased neonatal mortality.^5^ Teleneonatology, or video telemedicine used for newborn care, can connect experienced providers to newborn deliveries at hospitals without intensive care services. However, teleneonatology can be costly and requires abundant and ongoing resources.^6^ Additionally, prior simulation studies in other clinical contexts of cardiac arrest have not demonstrated an impact of telemedicine on parameters of cardiac resuscitation such as the no-flow fraction^7,8^ (the proportion of time during which patients do not receive chest compressions when clinically indicated). Therefore, further evidence that teleneonatal resuscitation improves neonatal outcomes is needed.

Multiple studies have evaluated the utility of telemedicine in cardiorespiratory resuscitation using simulation^7,9,10^ but the level of evidence is low. In a previous simulation study wherein pediatric residents and respiratory therapists were assigned but not randomized to either bedside resuscitation or video-assisted resuscitation facilitated by a neonatologist, the time to effective ventilation was decreased in the video-assisted resuscitation group.^9^ In addition, integration of telemedicine into clinical care is highly resource-dependent. It requires real-time, high-fidelity communication with a low failure rate at multiple sites as well as rapid connectivity given the time-sensitivity of neonatal resuscitation. Previous feasibility studies using teleneonatology reported audio and video quality unusable to nearly 20% of consultations,^11^ which are expected to improve with advancements in technology and internet access.^12^ Through a randomized simulation trial, we hypothesized that teleneonatal resuscitation as compared to routine resuscitation, would reduce the no-flow fraction.

## Methods

### Trial design and participants

This was a single-center, parallel, randomized trial conducted at the simulation center within Children’s of Alabama. Following Institutional Review Board approval, pediatric and internal medicine/pediatric residents were enrolled between November 2020 and January 2023. Trainees between years 1 and 4 were recruited by study personnel via email from the residency program at the University of Alabama at Birmingham/Children’s of Alabama and provided written consent before trial participation. Recruited trainees had to have previously completed The American Academy of Pediatrics Neonatal Resuscitation Program (AAP NRP) training, had at least four weeks of previous neonatal intensive care (NICU) exposure, and had 3 months elapsed since their previous NICU rotation. Participants were compensated with a $10 gift card.

### Simulated scenario and randomization

Prior to entering the simulation, participants were provided with the prompt: “You have been asked to attend the cesarean birth of Layla Thomas, a 25-week infant. The mother presented to the Labor & Delivery Unit one hour ago following a motor vehicle accident, no antenatal corticosteroids were given, and the baby will soon be delivered. Prepare for the delivery.”

**Fig. 1:**
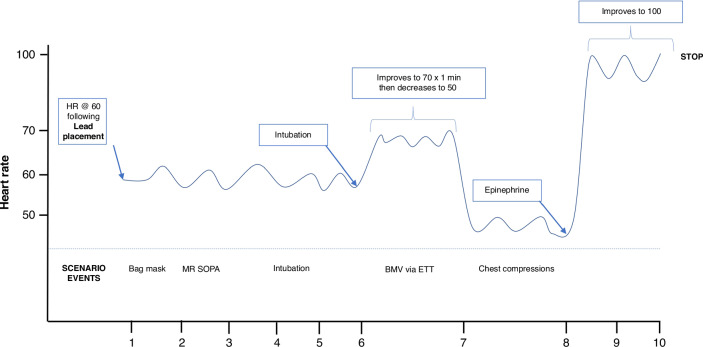
Changes in vital signs during the simulation. The noted changes in heart rate characteristics over time are indicated in response to participant interventions.

Within the simulated patient room were the manikin (Premature Anne^TM^, Laerdal Medical, Wappingers Falls, NY), hospital resuscitation cart (containing supplies for airway support, continuous monitoring, and thermoregulation), an Amwell C250 Telemedicine Cart (Boston, MA) adjacent to the bed, and two cameras to provide video recording for subsequent data analysis. So as to evaluate the no-flow fraction, the manikin in the scenario became bradycardic with a heart rate <60 following establishment of effective ventilation with additional time relevant details depicted in Fig. 1. During the first segment of the resuscitation, the heart rate remained between 60 and 100 and the participant received verbal feedback that there was no chest rise prompting the need for corrective bag-mask ventilation maneuvers. One minute after intubation the heart rate decreased to <60 for which chest compressions would be indicated. The heart rate would remain at 50 until endotracheal epinephrine was administered, the heart rate would then increase to >100, and the scenario would conclude. The scenario was stopped after a 10 min duration (given the anticipated ability to perform the simulation’s objectives within this time frame) or upon bradycardic resolution whichever occurred first. Both groups received a debrief at the resuscitation’s conclusion using the “debriefing with good judgement” model.^13^

Randomization was implemented using 1:1 ratio with a permuted-block randomization (with blocks of two and four) with the groups: teleneonatal resuscitation and routine resuscitation. Sequentially numbered, sealed opaque envelopes were used to conceal participant group allocation with computer-generated allocation sequences. Envelopes were opened just prior to participants’ entry into the simulation. Stratification was performed by post graduate year (PGY; PGY-1 and PGY-2/3/4). Participants could not be masked to intervention given the study design in which the teleneonatologist interacted with participants in the intervention arm. Study personnel assessing outcomes from video recordings were masked to randomization by elimination of audio recordings and the presence of the telemedicine cart within the visual field irrespective of randomization group.

### Intervention

After signing consent, participants completed a pre-simulation questionnaire indicating the level of training as well as experience with neonatal resuscitation. Those randomized to teleneonatal resuscitation also completed a post-simulation questionnaire with qualitative responses regarding the teleneonatal resuscitation. The same neonatologist was the facilitator for all participants randomized to teleneonatal resuscitation to minimize facilitator variability. The teleneonatologist was instructed to allow the participant to lead the resuscitation but to provide directive communication in instances wherein participants did not timely adhere to guidelines from the AAP NRP^1^ or failed to provide indicated interventions. The teleneonatologist only had access to information gained from visualization via the Amwell cart. The teleconnection occurred simultaneously with trainees’ entry into the simulated scenario. A research nurse assisted participants in the resuscitation by providing both nursing and respiratory therapy clinical responsibilities in the teleneonatal resuscitation group. Participants randomized to the routine resuscitation group were only supported by a research nurse. An additional research nurse present in both randomization groups became available to provide code epinephrine when requested by the participant.

### Study outcomes

Study measures considered in this investigation have demonstrably influenced patient outcomes or are a component to standardized resuscitation guidelines. The primary outcome was no-flow fraction defined as time without chest compressions divided by the time without spontaneous circulation when the heart rate was <60. Improvements in cardiopulmonary resuscitation inclusive of the no-flow fraction have been associated with a return of spontaneous circulation^14^ and survival.^15^

Secondary measures included aspects of respiratory support including the use of corrective measures to bag-mask ventilation (e.g., mask adjustment, repositioning the airway), the time at intubation, the frequency of intubation attempts, and the no blow-fraction. The no-blow fraction was calculated as the proportion of time an infant did not receive bag mask ventilation while the heart rate was <100. Additional secondary outcomes regarding cardiac resuscitation included time at which chest compressions were initiated, synchronization compliance, whether epinephrine was administered. All assessments were made using video recordings of participants’ performance masked to teleneonatologist presence.

Participants randomized to teleneonatal resuscitation completed a post-resuscitation questionnaire in which they indicated whether having a teleneonatologist improved the quality of resuscitation in addition to responses related to the audio and video quality and whether any issues with connectivity occurred. Free text comments were also provided.

### Power calculation

Simulated studies in other populations have reported a control group no-flow fraction of 0.19 (±0.10) in the setting of cardiopulmonary arrest.^8^ Using an alpha value of 0.05, 80% power, and an enrollment ratio of 1, a sample size of 50 participants would be needed to detect an 8% absolute risk difference (or 32% relative decrease) in the no-flow fraction. An additional 10 participants (5 per group) were recruited to account for the potential inability of video recordings to adequately assess for the primary outcome.

### Statistical analysis

Binary covariates were compared between groups using the Fisher exact test. For continuous measures, a Kolmogorov-Smirnov test of normality was performed after which the appropriate parametric or nonparametric test was conducted. In instances in which continuous data from only one randomization group were not normally distributed, data were log-transformed and then analyzed. No interim analyses were conducted during the study. The primary outcome was measured per intention-to-treat analysis. No adjusted analyses were performed on any study outcome. Analyses were performed using IBM SPSS Statistics Version 29.0 (Armonk, NY). A two-sided *p*-value < 0.05 was considered significant. For qualitative analyses research, personnel coded participant responses for specific themes with corresponding quotes.

## Results

**Fig. 2:**
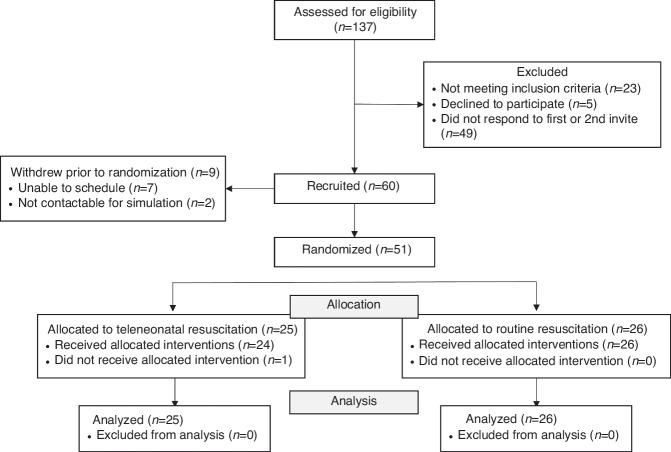
CONSORT flow diagram. Flow diagram depicts the number of participants excluded, recruited, and randomized during the trial.

One hundred thirty-seven trainees were assessed for study eligibility of which 77 (56%) were excluded for not responding to study invitation (*n* = 49), not meeting inclusion criteria (*n* = 23), or declining to participate (*n* = 5). Of the 60 participants recruited, 9 withdrew prior to randomization. Of the remaining participants, 25 were randomized to teleneonatal resuscitation and 26 were randomized to routine resuscitation (*n* = 51). One participant in the teleneonatal resuscitation group did not receive teleneonatal facilitation due to inability of the teleneonatologist to connect to the telemedicine cart. All participants were included in the analysis (Fig. 2).

Baseline characteristics, including PGY level, did not differ between randomization groups. Measures regarding resuscitation experience were similar between randomization groups. These measures included the number of neonatal resuscitations ever performed, the number of resuscitations performed in the prior 6 months, and the number of resuscitations in which bag-mask ventilation was required in the prior 6 months (Table 1).

**Table 1:** Baseline comparators between trainees randomized to teleneonatal and routine resuscitation.

	Teleneonatal resuscitation (*N* = 25)	Routine resuscitation (*N* = 26)	*P* value
Level of training			
PGY-1	6/25 (24)	5/26 (19)	0.74
PGY-2	10/25 (40)	12/26 (46)	0.60
PGY-3	8/25 (32)	9/26 (35)	1.00
PGY-4	1/25 (4)	0/26 (0)	0.49
NRP certified	25/25 (100)	26/26 (100)	1.00
NRP within the last 6 months	4/25 (16)	3/26 (12)	0.70
Prior neonatal resuscitations			
0	1/25 (4)	0/26 (0)	0.49
<10	1/25 (4)	4/26 (15)	0.35
10–20	9/25 (36)	3/26 (12)	0.05
20–30	5/25 (20)	7/26 (27)	0.74
>30	9/25 (36)	11/26 (42)	0.78
Resuscitations last 6 months			
0	8/25 (32)	4/26 (15)	0.20
<5	5/25 (20)	7/26 (27)	0.74
5–10	4/25 (16)	5/26 (19)	1.00
>10	8/25 (32)	9/26 (35)	1.00
BMV in the last 6 months			
0	4/25 (16)	5/26 (19)	1.00
<5	13/25 (52)	9/26 (35)	0.26
5–10	7/25 (28)	10/26 (39)	0.56
>10	1/25 (4)	1/26 (4)	1.00

*PGY* postgraduate year, *NRP* neonatal resuscitation program, *BMV* bag mask ventilation.

The primary outcome of no-flow fraction was significantly lower in the teleneonatal resuscitation group (0.06 ± 0.05) compared to the routine resuscitation group (0.07 ± 0.82; effect (95% CI) −16 (−43 to 0)). Regarding aspects of initial management, participants in the teleneonatal resuscitation group more frequently placed the manikin in a plastic wrap (96% vs 50%; RR (95% CI): 1.92 (1.30–2.84)) and placed electrocardiogram (ECG) leads earlier (35 vs 46 s; effect (95% CI): −13 (−25 to −2)). Other initial parameters including placement of ECG leads and the probe for pulse oximetry did not differ between groups (Table 2).

**Table 2:** Performance measures from the simulated resuscitation.

	Teleneonatal resuscitation (*N* = 25)	Routine resuscitation (*N* = 26)	Risk ratio (95% CI)
Primary outcome			
No flow fraction: median (IQR)	0.06 (0.05)	0.07 (0.82)	−16.3 (−43 to 0)^a*^
Initial management			
Placement in wrap (%)	24/26 (96)	13/26 (50)	1.9 (1.30–2.84)^*^
ECG leads placed (%)	24/25 (96)	21/26 (81)	1.2 (0.97–1.46)
Time to ECG leads (sec): median (IQR)	35 (16)	46 (37)	−13 (−25 to −2)^a*^
Sat probe placed (%)	24/25 (96)	25/26 (96)	1.0 (0.89–1.12)
Time of sat probe (sec): median (IQR)	45 (27)	41 (40)	5 (−10 to 15)^a^
Time of BMV (sec): mean (SD)	46 (14)	42 (18)	3.7 (−5.7 to 13)^b^
FiO_2_ increased (%)	23/25 (92)	18/26 (69)	1.3 (1.00–1.76)
Time of FiO_2_ increase (sec): median (IQR)	111 (191)	176 (602)	−22 (−199 to 45)^a^
MR SOPA			
Mask adjustment (%)	25/25 (100)	26/26 (100)	NS
Reposition airway (%)	25/25 (100)	23/26 (89)	NS
Suction mouth (%)	24/25 (96)	15/26 (58)	1.7 (1.19–2.34)^*^
Increase pressure (%)	23/25 (92)	12/26 (46)	2.0 (1.30–3.10)^*^
Advanced airway (%)	24/25 (96)	24/26 (92)	1.0 (0.91–1.19)
Correct MR SOPA sequence (%)	15/25 (60)	4/26 (15)	3.9 (1.50–10.2)^*^
Number of MR SOPA interventions (*n*): median (IQR)	5 (0)	4 (2)	1 (0–1)^a*^
Time at intubation (sec): median (IQR)	214 (113)	230 (139)	−16 (−67 to 43)^a^
Number of intubation attempts (*n*): median (IQR)	1 (1)	2 (1)	0 (−1 to 0)^a^
No blow fraction: median (IQR)	0.26 (0.06)	0.25 (0.09)	0.01 (−0.02 to 0.04)^a^
Cardiac resuscitation			
Chest compressions performed (%)	24/25 (96)	22/26 (85)	1.14 (0.95–1.36)
Time to compressions (sec): median (IQR)	318 (127)	267 (293)	57 (−44 to 138)^a^
Compression synchronization compliance (%)	24/25 (96)	20/26 (77)	1.25 (1.00–1.56)
Epinephrine given (%)	24/25 (96)	21/26 (81)	1.19 (0.97–1.46)
Time at epinephrine (sec): mean (SD)	395 (102)	444 (164)	−48.6 (−126 to 28)^b^
Time between chest compressions and epinephrine (sec): median (IQR)	56 (20)	223 (238)	−152 (−220 to −46)^a*^

*ECG* electrocardiogram, *IQR* interquartile range, *BMV* bag mask ventilation, *FiO*_*2*_ fraction of inspired oxygen, *MR SOPA* mask, reposition, suction, open mouth, pressure increase, alternate airway, *SD* standard deviation.

^a^Median difference (95% confidence interval).

^b^Mean difference (95% confidence interval).

^*^p-value < 0.05.

Performance of “MRSOPA” including mask adjustment, repositioning of the airway, suctioning the mouth, opening the mouth, increasing the pressure of bag-mask ventilation, and placing an advanced airway occurred in the correct sequence in a higher proportion of simulations in the teleneonatal resuscitation compared to the routine resuscitation group (60% vs 15%; RR (95% CI): 3.90 (1.50–10.2)). The time at intubation, the number of intubation attempts, and the no-blow fraction did not differ between groups (Table 2). Regarding cardiac resuscitation parameters, in addition to the no-flow fraction, the time between the initiation of chest compressions and epinephrine administration was shorter for the teleneonatal resuscitation (57 s) than the routine resuscitation groups (223 s; effect (95% CI): −152 (−220 to −46)). Time to chest compressions, chest compression synchronization compliance, and epinephrine administration did not differ between the groups (Table 2).

Participant responses from the teleneonatal resuscitation group indicated that having a teleneonatologist improved the quality of resuscitation in 96% (24 of 25) of simulated cases. Additionally, the audio and video quality were sufficient to help facilitate the resuscitation in 100% and 96% of simulated cases. Successful connection occurred upon the first attempt in 88% (22 of 25) of cases (Table 3). Qualitative themes identified from the prompt of providing any additional comments to the study team included appreciation of experienced guidance, a noted reduction in anxiety, and a sense of empowerment. Although the audio and video quality had been indicated as sufficient to help facilitate the resuscitation, technical challenges were also an identified theme (Table 4).

**Table 3:** Debriefing answers from teleneonatal resuscitation group.

	Strongly disagree	Disagree	Undecided	Agree	Strongly agree	Mean (SD)
Having a teleneonatologist improved the quality of resuscitation	0 (0)	0 (0)	1 (4)	1 (4)	23 (92)	4.9 (0.44)
The audio quality was sufficient to help facilitate the resuscitation	0 (0)	0 (0)	0 (0)	5 (20)	20 (80)	4.8 (0.41)
The video quality was sufficient to help facilitate the resuscitation	0 (0)	0 (0)	1 (4)	5 (20)	19 (76)	4.72 (0.54)
	Yes					
Was their successful connection on the first attempt?	22 (88)					
Was the connection ever lost or dropped?	0 (0)

**Table 4:** Qualitative themes and quotes from teleneonatal group debriefs.

Theme	Quote
Experience guidance	“Recommendations about next steps were priceless and incredibly helpful.” “Helped to make sure the (steps of) NRP were appropriate and nothing was forgotten.”
Reduction in anxiety	“…reduced a large amount of anxiety.” “It was very comforting to have a neonatologist…” “I was so relieved to have a teleneonatologist…” “…helpful in addressing a potentially chaotic situation.
Technical challenges	“Video camera malfunction. The neonatologist lost signal halfway.” “…some noise did drown out the neonatologist…” “Technical difficulties in terms of setting up the call.” “The volume may need to be louder in a more populated resuscitation.”
Empowerment	“I feel I could confidently resuscitate many more babies…” “I could focus on the hands on portion…” “I could focus on effectively performing the tasks.”

## Discussion

In this randomized, simulation trial, teleneonatal resuscitation reduced the no-flow fraction compared to routine resuscitation. Additionally, several metrics of resuscitation were also improved including the use of corrective measures for the delivery of bag-mask ventilation as well as the correct utilization of epinephrine following initiation of chest compressions.

The quality of chest compressions has been associated with survival in the adult literature. Many of these investigations have focused on out-of-hospital cardiac arrests, given that this is an environment in which variability in chest compression quality has been observed. In prior studies in out-of-hospital cardiac arrests, the quality of chest compressions inclusive of the time during which compressions were performed while indicated, has been associated with survival.^16,17^ For these reasons the American Heart Association’s guidelines for cardiorespiratory resuscitation recommend avoidance of unnecessary pauses while providing chest compressions,^18^ which remains critical to sustain cerebral and coronary perfusion.^19^ While the optimal no-flow fraction is not known, prior studies have reported improvement in survival when the no-flow fraction is below ~30%.^15^ In the present study, while the median no-flow fraction in the teleneonatal resuscitation (6%) resembled the routine resuscitation group (7%), we emphasize the widely different IQR, which was 5% in the teleneonatal resuscitation and 82% in the routine resuscitation group. Although resuscitation performance by routine resuscitation may adhere to NRP guidelines, teleneonatology may have the advantage of limiting variability between providers.

To our knowledge, no prior randomized simulation trial has evaluated the impact of teleneonatology on the no-flow fraction in neonatal resuscitation. However, in a prior study by in which pediatric trainees were assigned (but not randomized) to either video-assisted or routine neonatal resuscitation,^9^ video-assisted resuscitation was associated with a decrease in the time to effective ventilation as compared to routine resuscitation. This finding differs from the present study in that there was no difference in the time to intubation between groups. However, the study by Fang et al. may have been impacted by selection bias as participants were not randomized and more PGY-1 trainees were in the control group. Additionally, effective ventilation occurred at the completion of corrective measures (excluding intubation) in the video-assisted group but followed intubation in the control group. Both this and the present study documented more frequent use of corrective measures of bag-mask ventilation in the video-assisted resuscitation group. The observed similarities in no-flow fraction and time to intubation between groups suggest that pediatric trainees and early career pediatricians likely have sufficient experience in providing respiratory resuscitation, but may lack the skill set required for more acute, less frequent NRP measures.

Previous studies have tested the impact of telemedicine on the no-flow fraction in other simulated, clinical scenarios. In a multicenter trial of in-hospital cardiac arrest (*N* = 71),^8^ multidisciplinary participants were randomized to either control simulations or simulations assisted by a telemedical intensivist. In this trial, the no-flow fraction was similar between groups. However, this trial noted considerable challenges with audio quality which may have impacted two-way communication between the telemedical intensivist and participants. Audio fidelity has previously been cited as a considerable challenge in teleneonatal consultations.^11^ The audio and video quality, as reported by participants in the present study, was noted to be sufficient which may support differences in observed outcomes. In an additional, randomized trial of pediatric residents randomized either to Google Glass Assisted resuscitation or routine resuscitation (*N* = 42),^7^ the no-blow fraction was similar between groups. Similarly, this may have been attributable to challenges in communication, as cardiopulmonary resuscitation was interrupted more frequently in trainees randomized to Google Glass Assisted resuscitation. These findings highlight the importance of assessing audio-video performance in evaluations in evaluations of telemedicine with particular focus on the respective fidelity of specific devices.

Resuscitation education of providers in lower-level facilities may not be sufficient to reduce adverse perinatal outcomes. Implementation of the AAP NRP has previously shown a significant reduction in infant mortality.^20^ However, previous studies suggest that while NRP education may lead to initial improvement in education and training of healthcare providers, knowledge retention may not last beyond 3–6 months post-training and potentially less if deliveries occur infrequently.^21–23^ As recent exposure to neonatal resuscitation precluded eligibility in the present study, the performance of participants randomized to routine resuscitation may have been impacted by a decline in educational retention.

This randomized, simulation trial comparing teleneonatal to routine resuscitation has several strengths. Stratified randomization resulted in similar levels of training and exposure to neonatal resuscitation between randomization groups. Additionally, almost all of participants randomized to the teleneonatal resuscitation group noted that the audio and video quality were sufficient to facilitate the resuscitation, which previous randomized trials have noted as limitations.^7,8^ This study has several notable limitations. As the neonatologist in the simulated scenarios was aware of the clinical scenario, the no-flow fraction in trainees randomized to teleneonatal resuscitation may differ from the no-flow fraction observed in actual resuscitations. However, the observed no-blow fraction in the study approximates the fraction noted in prior studies.^24^ In addition, the findings in the study may be constrained to the technology utilized and may not be generalizable to all modalities of telemedicine. Lastly, the simulated scenario only included support from the participant and the research nurse, which may not accurately represent the resuscitation environment.

## Conclusion

In this randomized, simulation trial, teleneonatal resuscitation reduced the no-flow fraction compared to routine resuscitation. As the no-flow fraction has been implicated in adverse clinical outcomes in other patient populations,^14,15^ the use of teleneonatal resuscitation may improve neonatal outcomes. Additional evaluations of teleneonatology within diverse clinical environments and additional modalities are needed to further substantiate the value of this intervention.

## Supplementary information

CONSORT Checklist

## Data Availability

The data that support the findings of this study are available upon reasonable request to the corresponding author, S.J.G.

## References

[1] Weiner G. M., Zaichkin J., Kattwinkel J., American Academy of Pediatrics, American Heart Association. *Textbook of neonatal resuscitation*, 7th edition. edn. American Academy of Pediatrics: Elk Grove Village, IL, 2016.

[2] Chung, J. H. et al. Examining the effect of hospital-level factors on mortality of very low birth weight infants using multilevel modeling. *J. Perinatol.* **31**, 770–775 (2011). 21494232 10.1038/jp.2011.29

[3] Phibbs, C. S. et al. Level and volume of neonatal intensive care and mortality in very-low-birth-weight infants. *N. Engl. J. Med.* **356**, 2165–2175 (2007). 17522400 10.1056/NEJMsa065029

[4] Brantley, M. D., Davis, N. L., Goodman, D. A., Callaghan, W. M. & Barfield, W. D. Perinatal regionalization: a geospatial view of perinatal critical care, United States, 2010–2013. *Am. J. Obstet. Gynecol.* **216**, 185 e181–185 e110 (2017). 10.1016/j.ajog.2016.10.011 PMC11289569 27773712

[5] Shah, K. P., deRegnier, R. O., Grobman, W. A. & Bennett, A. C. Neonatal mortality after interhospital transfer of pregnant women for imminent very preterm birth in Illinois. *JAMA Pediatr.* **174**, 358–365 (2020). 32065614 10.1001/jamapediatrics.2019.6055 PMC7042951

[6] Thao, V. et al. Modeling the cost of teleneonatology from the health system perspective. *Telemed. J. e-Health* **28**, 1464–1469 (2022). 35235430 10.1089/tmj.2021.0527

[7] Drummond, D. et al. Google Glass for Residents dealing with pediatric cardiopulmonary arrest: a randomized, controlled, simulation-based study. *Pediatr. Crit. Care Med.* **18**, 120–127 (2017). 28165347 10.1097/PCC.0000000000000977

[8] Peltan, I. D. et al. Telemedical intensivist consultation during in-hospital cardiac arrest resuscitation: a simulation-based, randomized controlled trial. *Chest* **162**, 111–119 (2022). 35063451 10.1016/j.chest.2022.01.017 PMC9279650

[9] Fang, J. L., Carey, W. A., Lang, T. R., Lohse, C. M. & Colby, C. E. Real-time video communication improves provider performance in a simulated neonatal resuscitation. *Resuscitation* **85**, 1518–1522 (2014). 25132477 10.1016/j.resuscitation.2014.07.019

[10] Yang, C. P. et al. Can telemedicine improve adherence to resuscitation guidelines for critically Ill children at community hospitals? a randomized controlled trial using high-fidelity simulation. *Pediatr. Emerg. Care* **33**, 474–479 (2017). 26945195 10.1097/PEC.0000000000000653

[11] Fang, J. L. et al. Emergency video telemedicine consultation for newborn resuscitations: the mayo clinic experience. *Mayo Clin. Proc.* **91**, 1735–1743 (2016). 27887680 10.1016/j.mayocp.2016.08.006

[12] Haleem, A., Javaid, M., Singh, R. P. & Suman, R. Telemedicine for healthcare: capabilities, features, barriers, and applications. *Sens. Int.* **2**, 100117 (2021). 34806053 10.1016/j.sintl.2021.100117 PMC8590973

[13] Rudolph, J. W., Simon, R., Dufresne, R. L. & Raemer, D. B. There’s no such thing as “nonjudgmental” debriefing: a theory and method for debriefing with good judgment. *Simul. Health.* **1**, 49–55 (2006). 10.1097/01266021-200600110-00006 19088574

[14] Edelson, D. P. et al. Improving in-hospital cardiac arrest process and outcomes with performance debriefing. *Arch. Intern Med.* **168**, 1063–1069 (2008). 18504334 10.1001/archinte.168.10.1063

[15] Christenson, J. et al. Chest compression fraction determines survival in patients with out-of-hospital ventricular fibrillation. *Circulation* **120**, 1241–1247 (2009). 19752324 10.1161/CIRCULATIONAHA.109.852202 PMC2795631

[16] Gallagher, E. J., Lombardi, G. & Gennis, P. Effectiveness of bystander cardiopulmonary resuscitation and survival following out-of-hospital cardiac arrest. *JAMA* **274**, 1922–1925 (1995). 8568985

[17] Wik, L. et al. Why do some studies find that CPR fraction is not a predictor of survival? *Resuscitation* **104**, 59–62 (2016). 27155547 10.1016/j.resuscitation.2016.04.013

[18] Field, J. M. et al. Part 1: executive summary: 2010 American Heart Association guidelines for cardiopulmonary resuscitation and emergency cardiovascular care. *Circulation* **122**, S640–S656 (2010). 20956217 10.1161/CIRCULATIONAHA.110.970889

[19] Berg, R. A. et al. Adverse hemodynamic effects of interrupting chest compressions for rescue breathing during cardiopulmonary resuscitation for ventricular fibrillation cardiac arrest. *Circulation* **104**, 2465–2470 (2001). 11705826 10.1161/hc4501.098926

[20] Wegman, M. E. Annual summary of vital statistics-1989. *Pediatrics* **86**, 835–847 (1990). 2251021

[21] Singhal, N., McMillan, D. D., Yee, W. H., Akierman, A. R. & Yee, Y. J. Evaluation of the effectiveness of the standardized neonatal resuscitation program. *J. Perinatol.* **21**, 388–392 (2001). 11593374 10.1038/sj.jp.7210551

[22] Trevisanuto, D. et al. Knowledge gained by pediatric residents after neonatal resuscitation program courses. *Paediatr. Anaesth.* **15**, 944–947 (2005). 16238554 10.1111/j.1460-9592.2005.01589.x

[23] Carlo, W. A. et al. Educational impact of the neonatal resuscitation program in low-risk delivery centers in a developing country. *J. Pediatr.* **154**, 504–508 e505 (2009). 19058815 10.1016/j.jpeds.2008.10.005 PMC2909779

[24] Weidman, E. K., Bell, G., Walsh, D., Small, S. & Edelson, D. P. Assessing the impact of immersive simulation on clinical performance during actual in-hospital cardiac arrest with CPR-sensing technology: a randomized feasibility study. *Resuscitation* **81**, 1556–1561 (2010). 20724057 10.1016/j.resuscitation.2010.05.021

